# The predictive and prognostic role of metabolic and volume-based parameters of positron emission tomography/computed tomography as non-invasive dynamic biological markers in early breast cancer treated with preoperative systemic therapy

**DOI:** 10.3389/fonc.2022.976823

**Published:** 2023-01-04

**Authors:** Alessandro Inno, Marta Peri, Monica Turazza, Giuseppe Bogina, Alessandra Modena, Alberto Massocco, Modestino Pezzella, Matteo Valerio, Rosario Mazzola, Laura Olivari, Fabrizia Severi, Giovanni Foti, Cristina Mazzi, Fabiana Marchetti, Gianluigi Lunardi, Matteo Salgarello, Antonio Russo, Stefania Gori

**Affiliations:** ^1^ Medical Oncology Unit, IRCCS Ospedale Sacro Cuore Don Calabria, Negrar di Valpolicella (VR), Italy; ^2^ Medical Oncology Unit, Department of Surgical, Oncological and Stomatological Sciences, University of Palermo, Palermo, Italy; ^3^ Pathology Unit, IRCCS Ospedale Sacro Cuore Don Calabria, Negrar di Valpolicella (VR), Italy; ^4^ Breast Surgery Unit, IRCCS Ospedale Sacro Cuore Don Calabria, Negrar di Valpolicella (VR), Italy; ^5^ Radiation Oncology Unit, IRCCS Ospedale Sacro Cuore Don Calabria, Negrar di Valpolicella (VR), Italy; ^6^ Nuclear Medicine Unit, IRCCS Ospedale Sacro Cuore Don Calabria, Negrar di Valpolicella (VR), Italy; ^7^ Medical Physics Unit, IRCCS Ospedale Sacro Cuore Don Calabria, Negrar di Valpolicella (VR), Italy; ^8^ Radiology Unit, IRCCS Ospedale Sacro Cuore Don Calabria, Negrar di Valpolicella (VR), Italy; ^9^ Clinical Research Unit, IRCCS Ospedale Sacro Cuore Don Calabria, Negrar di Valpolicella (VR), Italy; ^10^ Clinical Analysis Laboratory and Transfusional Medicine, IRCCS Ospedale Sacro Cuore Don Calabria, Negrar di Valpolicella (VR), Italy

**Keywords:** early breast cancer, PET CT scan, SUV, preoperative chemotherapy, predictive factors

## Abstract

**Introduction:**

The role of fluorodeoxyglucose (FDG) positron emission tomography/computed tomography (PET/CT) in early breast cancer treated with preoperative systemic therapy (PST) is not yet established in clinical practice. PET parameters have aroused great interest in the recent years, as non-invasive dynamic biological markers for predicting response to PST.

**Methods:**

In this retrospective study, we included 141 patients with stage II-III breast cancer who underwent surgery after PST. Using ROC analysis, we set optimal cutoff of FDG-PET/CT parameters predictive for pathological complete response (pCR). We investigated the correlation between FDG-PET/CT parameters and pCR, median disease-free survival (DFS), and median overall survival (mOS).

**Results:**

At multivariable analysis, baseline SUVmax (high vs low: OR 9.00, CI 1.85 – 61.9, p=0.012) and Delta SUVmax (high vs low: OR 9.64, CI 1.84, 69.2, p=0.012) were significantly associated with pCR rates. Interestingly, we found that a combined analysis of the metabolic parameter Delta SUVmax with the volume-based parameter Delta MTV, may help to identify patients with pCR, especially in the subgroup of hormone receptor positive breast cancer. Delta SUVmax was also an independent predictive marker for both mDFS (high vs low: HR 0.17, 95%CI 0.05-0.58, p=0.004) and mOS (high vs. low: HR 0.19, 95%CI 0.04-0.95, p=0.029).

**Discussion:**

Our results suggest that Delta SUVmax may predict survival of early BC patients treated with PST.

## 1 Introduction

Breast cancer (BC) is the most common malignancy in women and represents a worldwide health problem. Therapy of early-stage disease includes local treatment with surgery, radiation therapy, or both, and systemic treatment with chemotherapy, endocrine therapy, biologic therapy, or combinations of the above. In general, chemotherapy regimens based on anthracyclines and taxanes reduce cancer-related mortality by about one-third in early-stage BC (1–3), by controlling undiscovered distant metastases. Among patients with HER2-positive early BC, the addition of anti-HER2 drugs to standard chemotherapy further improve the outcomes (4, 5).

Although the timing of chemotherapy in early-stage BC has not a demonstrated impact on survival (6–8), pre-operative systemic therapy (PST), also named neoadjuvant chemotherapy, is indicated in patients with locally advanced or inoperable breast cancer, desire of breast-conserving surgery, and operable tumors associated with a high likelihood of chemotherapy response, especially for tumors >2 cm (3, 9). The use of PST offers the advantage of downstaging the tumour and improving breast conservation rates, and provides an *in vivo* treatment response evaluation. Particularly, a pathological complete response (pCR) after PST is associated with favourable survival outcomes (10), especially for triple-negative breast cancer (TNBC) and with a lesser extent for HER2-positive BC. This does not apply to hormone receptor (HR)-positive, HER2-negative luminal subtypes, since pCR is rarely observed in these subtypes and patients maintain a good long-term prognosis independently of pCR (11). The administration of PST also represents an excellent research platform to test dynamic biological markers, such as metabolic parameters assessed through positron emission tomography (PET)/computed tomography (CT), or systemic inflammatory markers.

The role of PET/CT as non-invasive indicator of response has been recently established in early BC setting, since several meta-analyses showed that change of tumoral maximum standardized uptake value (Delta SUVmax) under therapy is significantly correlated with treatment response (12–14), and also with disease free-survival (DFS) and overall survival (OS) (15). More recently, PET/CT has been also used to optimize treatment in clinical trials, with the aim of discriminating good from poor responders to PST (16, 17).

Beyond Delta-SUVmax, other studies have focused on different metabolic parameters including tumor volume (MTV) and total lesion glycolysis (TLG), that evaluate not only metabolic activity but also the total tumor burden (18, 19). To date, however, the role of PET/CT for PST response evaluation is not fully established in clinical practice and no uptake cutoffs for pCR has been validated to classify the patients into metabolic responders and non-metabolic responders.

The aim of our study was to assess the predictive role of fluorodeoxyglucose (FDG) PET/CT indicators for pCR and survival outcomes, focusing on both metabolic and volume-based parameters.

## 2 Patients and methods

### 2.1 Patients

We included patients with histologically-proven, stage II-III breast cancer who underwent surgery after PST between January 2011 and May 2019 and performed FDG-PET/CT scan before starting PST at IRCCS Ospedale Sacro Cuore Don Calabria Institution. Last updated follow-up data was August 13th, 2021. We excluded patients with <=6 months follow-up. Clinicopathological characteristics, hematologic tests, nuclear medicine data (before and after PST) and outcome information were retrospectively collected and included into an anonymized database. The study was conducted in accordance with the Declaration of Helsinki and the International Conference on Harmonization Guidelines on Good Clinical Practice.

### 2.2 Study design

We retrospectively reviewed medical records of consecutive patients selected according to inclusion criteria. Data for this retrospective analysis were extracted from several sources, including Oncology Unit, Nuclear Medicine Unit and Pathology Unit database.

Cancer staging was reported in accordance with the 8th edition of the Union for International Cancer Control/American Joint Committee on Cancer (UICC/AJCC) TNM staging system.

The molecular subtype was evaluated considering a value of 20% as ki67 cutoff for differentiating Luminal A (ki67<=20%) and Luminal B HER2-negative (ki 67 >20%) BC.

The pCR was defined as the absence of invasive BC both in the breast and in axillar lymph-nodes at surgical specimen. Disease-free survival (DFS) is defined as the time from histological diagnosis to local/distant recurrence of tumor or death.

Overall survival (OS) was defined as time from diagnosis to last follow-up or death from any cause. Cutoffs value for metabolic parameters were calculated based on their ability to discriminate between pathological complete response and no response.

### 2.3 Nuclear medicine imaging analyses

All FDG-PET/CT scans were performed at the same Institution. To quantify 18F-FDG uptake, the tumoral standardized uptake values (SUVs) were measured.

We set the volume of interest (VOI) as the area in which FDG accumulated in the breast. The maximum value of SUV in the VOI was defined as the SUVmax, and the volume of voxels of >= 40% of the SUVmax in the VOI was defined as the MTV. The average SUV value in the voxel that showed >= 40% was defined as the SUVmean and TLG was defined as MTV x SUVmean.

### 2.4 Statistical analysis

Continuous variables were summarized using descriptive statistics, including number of observations, mean, standard deviation, median, and interquartile range. Categorical values were summarized using the number of observations and percentages. Data distribution was first assessed, and non-parametric tests applied accordingly afterwards. Statistical comparisons were made using Kruskal Wallis for continuous variables and Chi-Square test with simulated p-value for binary variables. The Spearman correlation coefficient was used to evaluate the linear correlation between two continuous variables.

The percentage changes (Delta%) of PET data and systemic inflammatory biomarkers at baseline and after PST were calculated as follows: percentage change (Delta%) = (delayed parameter - baseline parameter)/baseline parameter x 100. The optimal cutoff point for the PET parameters (SUVmax, MTV, TLG, Delta SUVmax, Delta MTV, Delta TLG) was obtained using the maximum sum of sensitivity and specificity (Youden’s J statistic) considering pCR outcome as reference standard. PET parameters were then dichotomized into “low” or “high” values based on the aforementioned threshold. Demographic and clinical characteristic along with PET parameters, were analysed by univariable logistic regression models to explore their association with the likelihood of reaching a pCR. Only variables significantly associated (p-value < 0.2) to a pCR were included in the full logistic regression model. Backward and forward elimination based on AIC was used for final model selection. The variable describing molecular subtypes was excluded from the logistic regression model because no pathological complete responses were observed in the luminal A and lumimal B (HER2+) subgroups. Model-building strategies included checking for convergence, correlation, and goodness-of-fit test. The Kaplan Meier method and log-rank test were performed to assess the difference in survival probabilities between type of pathological response and other covariates of interest. The multivariate Cox proportional hazards model was used to estimate the hazard ratios and 95% CIs for OS and DFS after checking for proportional hazard assumption.

All statistical tests were performed using two-sided 5% significance levels and P <.05 was considered statistically significant. Statistical analysis was performed using R statistical software version 4.1.1 (R Core Team 2021).

## 3 Results

### 3.1 Characteristics of the whole study population

The analysis included 141 patients with diagnosis of stage II-III breast cancer who underwent surgery after PST. Detailed demographics and clinical characteristics are reported in **Table 1**. Median age at diagnosis was 48 years, with an interquartile range (IQR) from 43 to 60 years. Prevalent histology was invasive ductal carcinoma (89.4%) and most cases were premenopausal (64.5%), cT2 (80.1%) and N1 (61.0%) at the diagnosis. Only 2.1% of tumors had a low grade (G1) and median Ki67 proliferation index was 30% (IQR 20-50) at diagnosis. Patients with HER2-enriched molecular subtype (i.e. HR-negative, HER2-positive) were 24.1%, Luminal B/HER2-negative were 23.4%, Luminal B/HER2-positive and triple-negative were 17.0% each, and Luminal A tumors were 16.3% of cases. One hundred and thirty-six patients (96.5%) received an anthracycline-based chemotherapy, and 41.1% received trastuzumab in combination with chemotherapy. After PST, 93 patients (66.4%) underwent a conservative surgical approach, while the remaining 47 (33.6%) were treated by mastectomy.

**Table 1:** Demographics and clinical characteristics of whole population related to pCR.

			Pathological Response		
	N	Overall	No response or partial	Complete	
		***(N=141)***	***(N=106)***	***(N=32)***	
**Age**	141	48 [43 - 60]	48 [42 - 60]	48 [43 - 60]	P=0.742^†^
**Post -menopausal**	141	50 (35.5)	36 (34.0)	14 (43.8)	P=0.400^§^
**Clinical T stage (cT)**	141				P=0.587^§^
** T0**		0 (0.0)	0 (0.0)	0 (0.0)	
** T1**		8 (5.7)	6 (5.7)	2 (6.2)	
** T2**		113 (80.1)	85 (80.2)	26 (81.2)	
** T3**		14 (9.9)	9 (8.5)	4 (12.5)	
** T4**		6 (4.3)	6 (5.7)	0 (0.0)	
**Clinical N stage (cN)**	141				P=0.288^§^
** N0**		34 (24.1)	26 (24.5)	8 (25.0)	
** N1**		86 (61.0)	67 (63.2)	16 (50.0)	
** N2**		17 (12.1)	11 (10.4)	6 (18.8)	
** N3**		4 (2.8)	2 (1.9)	2 (6.2)	
**Stage**	141				P=0.679^§^
** 2A**		29 (20.6)	22 (20.8)	7 (21.9)	
** 2B**		77 (54.6)	60 (56.6)	15 (46.9)	
** 3A**		13 (9.2)	8 (7.5)	5 (15.6)	
** 3B**		12 (8.5)	8 (7.5)	3 (9.4)	
** 3C**		10 (7.1)	8 (7.5)	2 (6.2)	
**Baseline grading**	140				P=0.003^§^
** 1**		3 (2.1)	3 (2.8)	0 (0.0)	
** 2**		49 (34.8)	45 (42.5)	3 (9.4)	
** 3**		88 (62.4)	57 (53.8)	29 (90.6)	
** Missing**		1 (0.7)	1 (0.9)	0 (0.0)	
**Hystotipe**	139				P=0.262^§^
** Ductal**		126 (89.4)	92 (86.8)	31 (96.9)	
** Lobular**		9 (6.4)	9 (8.5)	0 (0.0)	
** Others**		4 (2.8)	3 (2.8)	1 (3.1)	
** Missing**		2 (1.4)	2 (1.9)	0 (0.0)	
**Molecular subtype**	138				P<0.001^§^
** LUM-A**		23 (16.3)	23 (21.7)	0 (0.0)	
** LUM-B (HER2-)**		33 (23.4)	31 (29.2)	0 (0.0)	
** LUM-B (HER2+)**		24 (17.0)	16 (15.1)	8 (25.0)	
** HER2+**		34 (24.1)	16 (15.1)	17 (53.1)	
** TN**		24 (17.0)	17 (16.0)	7 (21.9)	
** Missing**		3 (2.1)	3 (2.8)	0 (0.0)	
**Baseline SUV_max_**	141	10.1 [5.9; 14.7]	8.8 [5.4; 12.8]	13.2 [9.9; 20.2]	P=0.002^†^
**Delta SUV_max_ %**	138	-68.2 [-100.0; -30.4]	-57.6 [-79.1; -22.9]	-100.0 [-100.0; -100.0]	P<0.001^†^
**Baseline MTV**	141	3.88 [2.13; 8.27]	4.12 [2.19; 8.57]	3.11 [1.64; 7.37]	P=0.244^†^
**Delta MTV %**	139	-89 [-100; -45]	-73 [-100; -36]	-100 [-100; -100]	P<0.001^†^
**Baseline TLG**	118	23.0 [11.6; 65.1]	26.8 [10.7; 61.4]	16.8 [11.6; 85.0]	P=0.793^†^
**Delta TLG %**	115	-88.0 [-100.0; -68.9]	-84.9 [-100.0; -56.6]	-100.0 [-100.0; -98.1]	P<0.001^†^

N is the number of non-missing value. Continuous variables are expressed as: median [Q1-Q3]. Categorical variables as n (%). ^†^Wilcoxon-Mann-Whitney test. ^§^Chi-square test.

Thirty-two patients (23.2%) obtained a pCR after PST. At data cutoff, number of deaths from any cause were 23/141 and median OS was 57.3 months (interquartile range 41.9-83.0) for the whole population. Overall relapses were 39/141, of which 29 were distant and 10 were local, with a median DFS of 52 months (IQR 36.8-77.9 months).

Luminal-A, Luminal-B HER2-negative, Luminal-B HER2-positive, HER2 enriched and TNBC had a mDFS of 73.8 [48.0-96.5], 41.2 [32.6-59.5], 58.9 [41.9-82.4], 52.5 [35.9-74.7] and 45.9 [34.3–73.1] months, respectively.

Luminal-A, Luminal-B HER2-negative, Luminal-B HER2-positive, HER2 enriched and TNBC had a mOS of 81.4 [70.7-96.6], 47.7 [34.9-66.5], 61.0 [42.7-85.7], 54.7 [45.6-77.7] and 54.9 [41.2–73.2] months, respectively.

Time to relapse distribution according to molecular subtype showed a peak after primary resection between 36 and 47 months. Luminal A and HER2-positive patients experienced later relapse as compared with the other molecular subtypes. Bone (14.9%), liver (12.8%), extra-regional lymph nodes (7.4%), and lung (6.4%) were the most common sites of relapse.

### 3.2 Relationships between clinicopathological factors and pCR

Association between clinicopathological characteristics and pCR are reported in **Table 1**. The descriptive analysis showed that pCR rates were significantly associated with grading (p<0.001) and molecular subtype (p<0.001). In particular, none of the patients with Luminal A or Luminal B HER2-negative did achieve a pCR. The pCR rate was higher in HER2-enriched patients (53.1%), followed by Luminal B HER2-positive (25%) and triple-negative (21.9%).

We explored the association between systemic inflammatory indicators and pCR rate without discovering any relevant associations (**Supplementary Table 1**). Even correlations between inflammation biomarkers and metabolic parameters did not show any significant association (data not shown).

### 3.3 Relationships between metabolic parameters and pCR

Of the baseline metabolic parameters, only tumoral SUVmax as continuous variable showed a significant association with pCR (p<0.001). The pCR was significantly associated with higher Delta SUVmax, Delta MTV and Delta TLG (p<0.001), suggesting a predictive role for changes of both metabolic (SUV) and volume-based parameters (MTV and TLG) after treatment as compared with baseline.

### 3.4 Determination of the optimal metabolic parameters cutoff values for pCR

The cutoff values of baseline metabolic parameters and their reduction rate are shown in **Table 2**. We set the optimal cutoff values of baseline metabolic parameters for SUVmax, MTV, and TLG at 9.2, 1.5 and 16.9, respectively. For dynamic parameters the optimal cutoffs of Delta SUVmax, MTV, and TLG were -98.3%, -84.2%, and -94.4%, respectively.

**Table 2:** Optimal Metabolic Parameters Cutoff Values for pCR.

Variable	Cutoff	Sensibility(%)	Specificity(%)
Baseline SUV_max_	9.2	81.2	53.8
Baseline MTV	1.5	25.0	92.5
Baseline TLG	16.9	52.0	60.0
Delta SUV_max_ %	-98.3	80.6	88.7
Delta MTV %	-84.2	90.6	57.5
Delta TLG %	-94.4	84.0	68.5

### 3.5 Univariable and multivariable analyses of pCR

Among clinicopathological characteristics, logistic regression univariate model showed higher pCR rates for grade 3 compared to grade 2 (p=0.002) (**Supplementary Table 2**). Among metabolic parameters, baseline SUVmax (high *vs* low) (p<0.001) and MTV (high *vs* low) (p=0.010), Delta SUVmax (high *vs* low) (p<0.001), Delta TLG (high *vs* low) (p<0.001) and Delta MTV (high *vs* low) (p<0.001) were significantly associated to pCR. At multivariate analysis, baseline grading (3 *vs* 2) (OR 17.2, CI 2.39 - 372, p=0.017), baseline SUVmax (high *vs* low) (OR 9.00, CI 1.85 – 61.9, p=0.012) and Delta SUVmax (high *vs* low) (OR 9.64, CI 1.84, 69.2, p=0.012) were significantly associated to pCR (**Table 3**).

**Table 3:** Multivariable logistic regression model for pCR.

Characteristic	OR*^1^*	95% CI*^1^*	p-value
Baseline grading			
2	—	—	
3	17.2	2.39, 3.72	**0.017**
Baseline SUV_max_			
Low	—	—	
High	9.00	1.85, 61.9	**0.012**
Baseline MTV			
Low	—	—	
High	0.15	0.02, 1.31	0.087
Delta SUV_max_			
Low	—	—	
High	9.64	1.84, 69.2	**0.012**
Delta TLG			
Low	—	—	
High	4.84	0.73, 33.9	0.10
No. Obs.	111		

^1^OR, Odds Ratio, CI, Confidence Interval. Bold values indicate a p value of <.05.

### 3.6 Predictive role of combined Delta SUVmax and Delta MTV for pCR

Three subgroups of patients were obtained by combining dichotomized Delta SUVmax and Delta MTV, respectively: “High-High”, “Low-Low” and “Low-High” subgroups. Very few patients had high Delta SUVmax associated with low Delta MTV.

**Table 4** describes the relationship between combined Delta SUVmax/Delta MTV and pCR, highlighting that a low Delta SUVmax is related to a decreased pCR rate. Of interest, the intermediate subgroup “Low-High” showed a higher pCR rate compared to “Low-Low”.

**Table 4:** Association between combined Delta SUVmax and Delta MTV evaluation and baseline clinicopathological characteristics and pCR.

Delta SUV_max_ x Delta MTV					
	N	High.High	Low.High	Low.Low	Test Statistic
		***(N=36)***	***(N=38)***	***(N=63)***	
Patological response after PST	138				P<0.001^§^
No response or partial		11 (30.6)	34 (89.5)	60 (95.2)	
Complete		25 (69.4)	3 (7.9)	3 (4.8)	
Missing		0 (0.0)	1 (2.6)	0 (0.0)	

^§^Chi-square test.

### 3.7 Relationships between clinicopathological factors, including metabolic parameters, and survival

**Figure 1:**
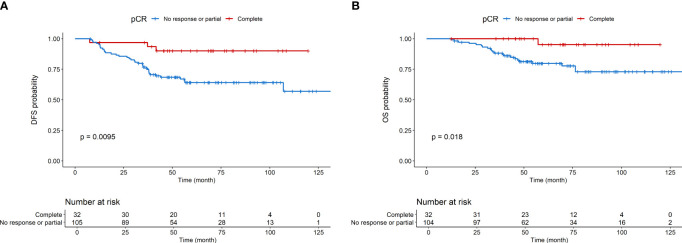
Disease-free survival **(A)** and overall survival **(B)** according to pCR.

**Figure 2:**
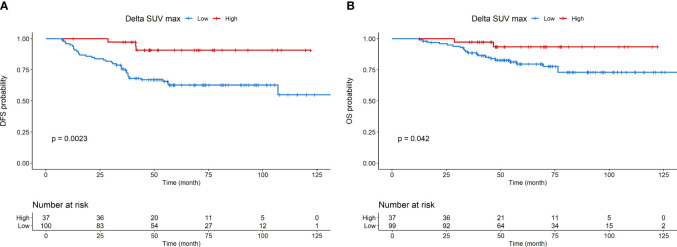
Disease-free survival **(A)** and overall survival **(B)** according to Delta SUVmax.

**Figure 3:**
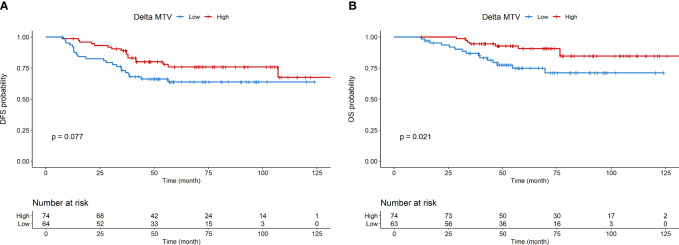
Disease-free survival **(A)** and overall survival **(B)** according to Delta MTV.

**Figure 4:**
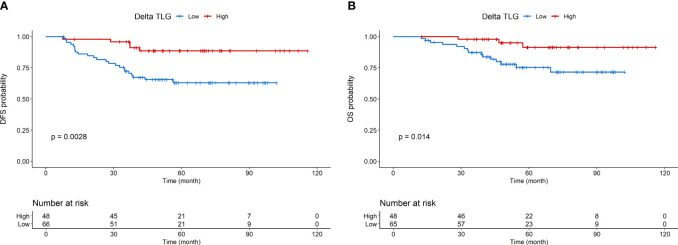
Disease-free survival **(A)** and overall survival **(B)** according to Delta TLG.

Kaplan-Meier curves for DFS and OS are shown in **Figures 1A, B**, respectively. Kaplan-Meier curves for OS according to molecular subtype are shown in **Supplementary Figures 1, 2**. Detailed association between clinicopathological characteristics and metabolic parameters and clinical outcomes are reported in **Figures 2**–**4** and **Supplementary Tables 3, 4**.

At multivariable analysis, clinical stage at diagnosis (III *vs* II: HR 1.98, 95%CI 1.01 – 3.87, p=0.046) and Delta SUVmax (high *vs* low: HR 0.17, 95%CI 0.05-0.58, p=0.004) were associated with DFS, whereas grading (Grade 3 *vs* 2: HR 2.81, 95%CI 1.06-8.07, p=0.038), age (HR 1.04, 95%CI 1.00-1.08, p=0.27) and Delta SUVmax (high *vs*. low: HR 0.19, 95%CI 0.04-0.95, p=0.029) to OS (**Tables 5**, **6**). This final selected model did not include pCR, since this variable was excluded through the stepwise model selection.

**Table 5:** Multivariable Cox proportional hazard model of the clinicopathological characteristics and metabolic parameters for DFS.

Characteristic	HR*^1^*	95% CI*^1^*	p-value
**Stage**			
2A 2B	—	—	
3A 3B 3C	1.98	1.01, 3.87	**0.046**
**Baseline grading**			
2	—	—	
3	2.78	1.30, 5.97	**0.009**
**Baseline SUV_max_**			
Low	—	—	
High	0.57	0.29, 1.12	0.10
**Delta SUV_max_**			
Low	—	—	
High	0.17	0.05, 0.58	**0.004**
No. Obs.	133		

^1^ HR, Hazard Ratio, CI, Confidence Interval. Bold values indicate a p value of <.05.

**Table 6:** Multivariable Cox proportional hazard model of the clinicopathological characteristics and metabolic parameters for OS.

Characteristic	HR*^1^*	95% CI*^1^*	p-value
Baseline grading			
2	—	—	
3	2.93	1.06, 8.07	**0.038**
**Age**	1.04	1.00, 1.08	**0.027**
Delta SUV_max_			
Low	—	—	
High	0.19	0.04, 0.84	**0.029**
No. Obs.	132		

^1^ HR, Hazard Ratio, CI, Confidence Interval. Bold values indicate a p value of <.05.

## 4 Discussion

Preoperative systemic therapy (PST) in breast cancer (BC) represents an intriguing research topic. Consistently with other reports, in the present study we observed at univariable analyses that pCR is significantly associated with DFS and OS among BC patients treated with PST.

Many efforts are ongoing to improve PST in order to obtain higher rates of pCR. Recent advances in PST include the use of dual anti-HER2 blockade with trastuzumab plus pertuzumab associated with chemotherapy in HER2-positive (Neosphere, TRYPHAENA and PEONY trial) (20–22) and the addition of platinum compounds in TNBC (23). The identification of patients with pCR after PST also provides the opportunity of tailoring postoperative treatments. At this regard, two randomized trials have recently changed the clinical practice, in TNBC (24) and in HER2-positive BC (25). Multiple trials are exploring the use of immune-checkpoints inhibitors in TNBC (A-brave, NCT02954874, KEYNOTE-522) and CDK4/6 inhibitors in hormone receptor-positive BC (Monarch-e). Poly (ADP‐ribose) polymerase (PARP) inhibitors have recently emerged as a promising class of therapeutics in BC, and several clinical studies in early stage are ongoing (26).

Although pCR has a relevant prognostic impact at individual level, it seems to be not a surrogate endpoint for both DFS and OS at trial level (27). In our study, in fact, pCR was excluded from the final multivariable analysis during the stepwise elimination process because its association with survival outcomes was weak. Therefore, other predictive factors for survival should be investigated in the setting of PST in early BC (20–23).

In the last years, some retrospective studies have evaluated the predictive role of metabolic parameters in BC (18, 19, 28). In the present study we focused on evaluation of the predictive role of PET/TC parameters as non-invasive dynamic biomarkers after PST. Although baseline SUVmax, baseline MTV, Delta SUVmax, Delta TLG and Delta MTV were significantly associated to pCR at univariable analysis, only baseline SUVmax and Delta SUVmax maintained an independent role for predicting pCR at multivariable analysis. This observation is consistent with other published studies (12–14).

In our study, patients with “low” Delta SUVmax achieved low pCR rates. Patients with “low” Delta SUVmax, however, were a heterogeneous population in terms of molecular subtype, mostly characterized by high HR expression, which is typically associated with chemoresistance. Since Delta SUVmax and Delta MTV were both significantly associated with pCR at univariable analysis, we further analyzed the role of a combination of these two metabolic parameters for predicting pCR. The combination of the two allows to assess metabolic and volume-based parameters together. Interestingly, we observed higher pCR rate in the “low” Delta SUVmax/”high” Delta MTV subgroup compared to “low” Delta SUVmax/”low” Delta MTV subgroup, suggesting that MTV together with SUVmax could be a useful dynamic biomarker for pCR in clinical practice, especially in heterogeneous breast cancer subtypes such as those HR-positive.

Recent reports described an interesting association between PET uptake and different biomarkers of inflammation (NLR, PLR, SIRS) (29–32), noting that patients with high SUVmax and low NLR (indicating a status of immune system activation) had lower recurrent disease after surgery (30). In the present study, however, we did not find any correlation between PET parameters and systemic biomarkers of inflammation. An explanation for these conflicting results among studies could be that systemic inflammatory biomarkers could not reliably mirror the inflammatory status of tumor microenvironment, possibly related to metabolic parameters of the tumor detected through the PET/CT.

Interestingly, we reported a significant association at multivariable analysis between Delta SUVmax and survival outcomes, both in terms of DFS and OS, and this is consistent with the results of a meta-analysis that showed a significant predictive value of Delta SUVmax for disease recurrence and survival (12). Taken together, these data may support the rationale for including PET/CT assessment before, interim and after treatment in clinical trials on PST for early BC.

Our study has some limitations due to the retrospective design, the small sample size, and the heterogeneity of the study population in terms of molecular subtypes and treatment. Considering the prognosis of early BC, an extended follow-up period may provide additional information with the aim of identify patients that may need additional tailored treatments. Larger studies on single molecular subtypes may provide further information on the role of PET/CT among patients with early BC receiving PST.

## 5 Conclusion

The present study suggested a role for PET/TC imaging as non-invasive dynamic biomarker in early BC treated with PST. Particularly, Delta SUVmax was significantly associated with pCR, DFS and OS, and possibly deserve further investigation in prospective neoadjuvant trials as potential surrogate endpoint for survival.

Interestingly, this study is the first attempt to evaluate the prognostic role of volume-based parameters in BC neoadjuvant setting. Particularly, our results suggest that a combined evaluation of Delta SUV and Delta MTV could help to refine prognosis, especially among patients with HR-positive tumors.

## Data availability statement

The raw data supporting the conclusions of this article will be made available by the authors, without undue reservation.

## Ethics statement

The studies involving human participants were reviewed and approved by Ethics Committee (Comitato Etico per la sperimentazione clinica delle province di Verona e Rovigo). Written informed consent for participation was not required for this study in accordance with the national legislation and the institutional requirements.

## Author contributions

SG designed the study. MT, AMo, GB, AMa, MPer, MPez, RM, LO, MS, FS and GF collected data. MV and CM analyzed data. MPer, CM and AI wrote the draft. AI, SG and AR critically revised the manuscript. All authors contributed to the article and approved the submitted version.
