# Supplementary material for: The predictive and prognostic role of metabolic and volume-based parameters of positron emission tomography/computed tomography as non-invasive dynamic biological markers in early breast cancer treated with preoperative systemic therapy
Source: Front Oncol. 2023 Jan 4;12:976823. doi: 10.3389/fonc.2022.976823 (PMC9846157; doi:10.3389/fonc.2022.976823)
Supplement: Supplementary file 1 [file DataSheet_1.pdf]

Supplementary Material

1 Supplementary Figures

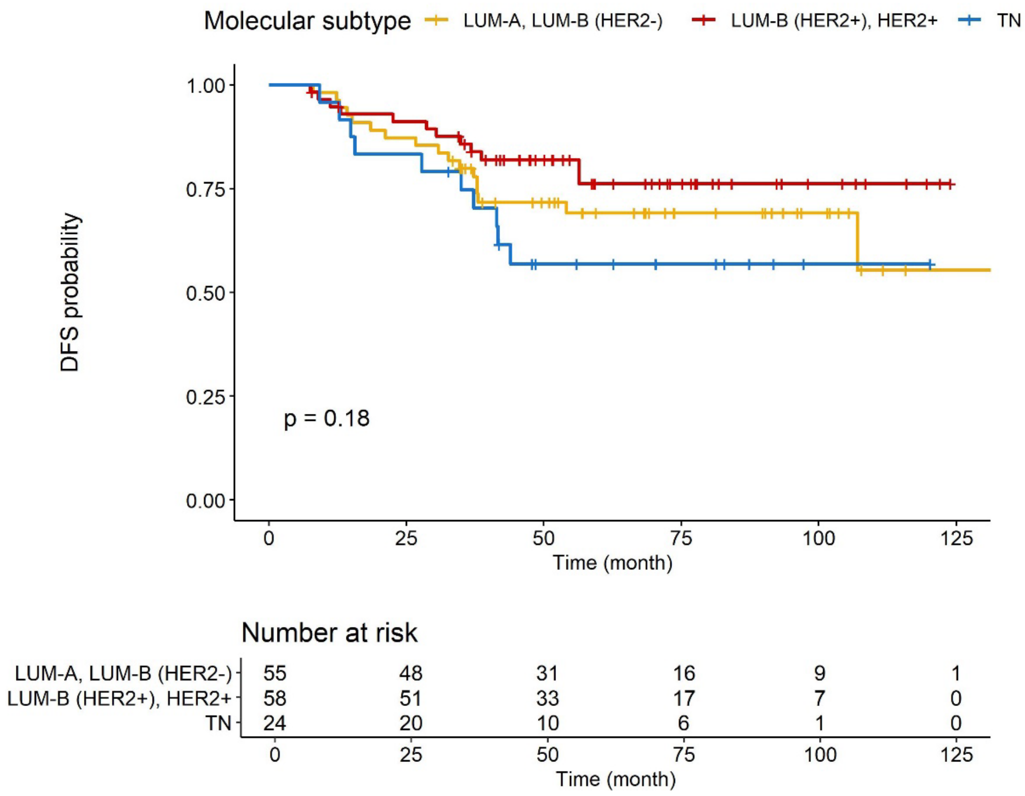

Supplementary Figure 1. Distant relapse-free survival according to molecular subtypes

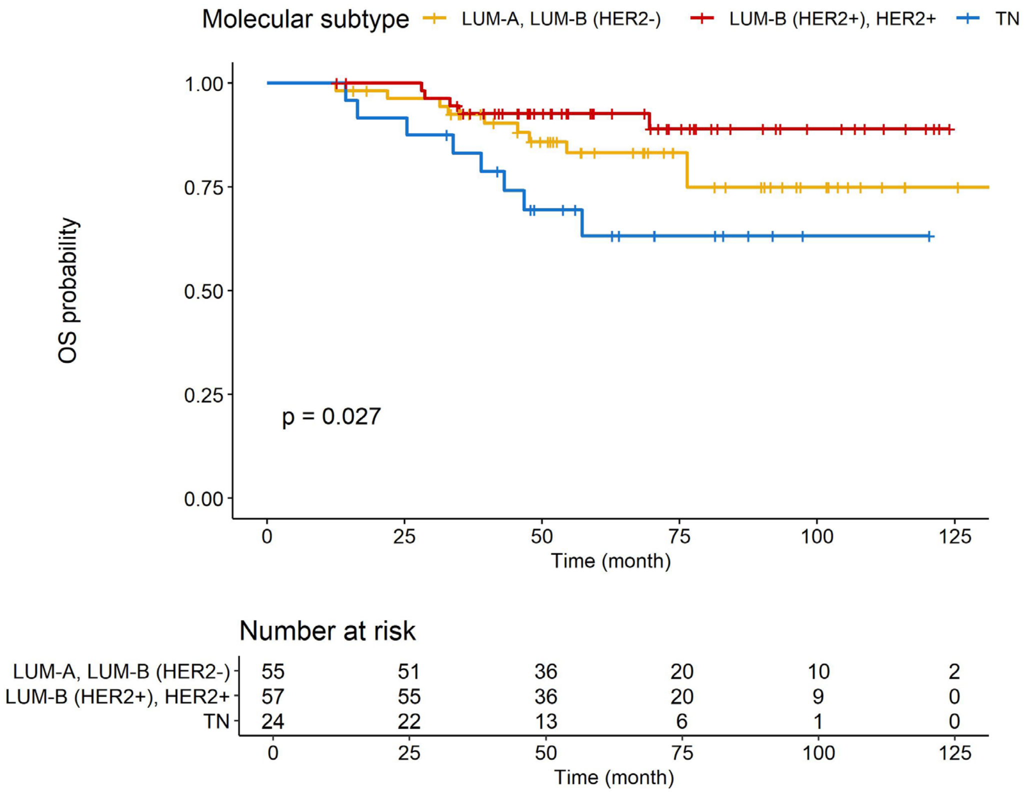

Supplementary Figure 2. Overall survival according to molecular subtypes

2 Supplementary Tables

Supplementary Table 1 Relative change from baseline of systemic inflammatory biomarkers.

	pCR		
	No response or partial *N=106*	Complete *N=32*	Test Statistic
Delta PMR	-13.8 [-26.3 - 12.0]	2.3 [-25.1 - 14.2]	P=0.290†
Delta NMR	-23.3 [-37.9 - -2.0]	-22.0 [-47.2 - 2.5]	P=0.957†
Delta PLR	40.6 [13.5 - 82.7]	25.5 [6.5 - 73.2]	P=0.405†
Delta NLR	22.6 [-7.0 - 72.6]	4.8 [-17.4 - 36.1]	P=0.087†
Delta LMR	-37.8 [-52.9 - -19.8]	-30.4 [-41.8 - 0.7]	P=0.073†

N is the number of non-missing value. Continuous variables are expressed as: median [IQR]. †Wilcoxon-Mann-Whitney test.

pCR: pathological Complete Response, PMR: platelet-to-monocyte ratio, NMR: neutrophil-to-monocyte ratio, PLR: platelet-to-lymphocyte ratio, NLR: neutrophil-to-lymphocyte ratio, LMR: lymphocytes-to-monocytes ratio.

Supplementary Table 2. Univariable logistic regression models for pCR.

Characteristic	N	OR[1]	95% CI[1]	p-value
**Age**	138	0.99	0.96, 1.03	0.642
**Menopausal status**	138			
Pre-menopause		—	—	
Post-menopause		1.51	0.67, 3.38	0.314
**Stage**	138			
2A 2B		—	—	
3A 3B 3C		1.55	0.63, 3.67	0.324
**Baseline grading**	134			
2		—	—	
3		7.63	2.50, 33.3	**0.001**
**Ki67 cutoff 20**	134			
<=20		—	—	
>20		2.36	0.89, 7.46	0.107
**Baseline SUV max**	138			
Low		—	—	
High		5.04	2.03, 14.4	**0.001**
**Baseline MTV**	138			
Low		—	—	
High		0.24	0.08, 0.73	**0.010**
**Baseline TLG**	115			
Low		—	—	
High		0.62	0.25, 1.50	0.285
**Delta SUV max**	137			
Low		—	—	
High		32.6	11.8, 104	**<0.001**
**Delta TLG**	114			

Characteristic	N	OR[1]	95% CI[1]	p-value
Low		—	—	
High		11.4	3.93, 42.0	**<0.001**
**Delta MTV**	138			
Low		—	—	
High		13.1	4.31, 57.1	**<0.001**

[1] OR = Odds Ratio, CI = Confidence Interval

Supplementary Table 3. Univariable Cox proportional hazard models of the clinicopathological characteristics and metabolic parameters for DFS

Characteristic	N	HR[1]	95% CI[1]	p-value
**Age**	140	1.01	0.98, 1.04	0.460
**Patological complete response**	136			
No response or partial		—	—	
Complete		0.24	0.07, 0.78	**0.017**
**Menopausal status**	140			
Pre-menopause		—	—	
Post-menopause		1.58	0.84, 2.97	0.153
**Molecular subtype**	137			
LUM-A, LUM-B (HER2-)		—	—	
LUM-B (HER2+), HER2+		0.65	0.31, 1.37	0.260
TN		1.43	0.65, 3.12	0.373
**Ki67 cutoff 20**	136			
<=20		—	—	
>20		1.38	0.67, 2.86	0.384
**Stage**	140			
2A 2B		—	—	
3A 3B 3C		1.87	0.97, 3.60	**0.061**
**Baseline grading**	136			
2		—	—	
3		1.80	0.87, 3.74	0.112
**Baseline SUV max**	140			
Low		—	—	
High		0.61	0.32, 1.16	0.131
**Baseline MTV**	140			
Low		—	—	

Characteristic	N	HR[1]	95% CI[1]	p-value
High		1.73	0.53, 5.63	0.363
**Baseline TLG**	117			
Low		—	—	
High		1.53	0.71, 3.29	0.278
**Delta TLG**	114			
Low		—	—	
High		0.26	0.10, 0.67	**0.006**
**Delta SUV max**	137			
Low		—	—	
High		0.19	0.06, 0.63	**0.006**
**Delta MTV**	138			
Low		—	—	
High		0.57	0.30, 1.07	0.081

[1] HR = Hazard Ratio, CI = Confidence Interval

Supplementary Table 4 Univariable Cox proportional hazard models of the clinicopathological characteristics and metabolic parameters for OS

Characteristic	N	HR[1]	95% CI[1]	p-value
**Patological complete response**	136			
No response or partial		—	—	
Complete		0.13	0.02, 0.96	**0.045**
**Age**	139	1.03	1.00, 1.07	0.074
**Menopausal status**	139			
Pre-menopause		—	—	
Post-menopause		2.05	0.90, 4.66	0.086
**Molecular subtype**	136			
LUM-A, LUM-B (HER2-)		—	—	
LUM-B (HER2+), HER2+		0.48	0.16, 1.40	0.179
TN		2.01	0.79, 5.09	0.142
**Ki67 (%)**	135			
<=20		—	—	
>20		2.48	0.84, 7.32	0.101
**Stage**	139			
2A 2B		—	—	
3A 3B 3C		0.95	0.35, 2.56	0.918
**Baseline grading**	135			
2		—	—	
3		2.13	0.78, 5.82	0.141
**Baseline SUV max**	139			
Low		—	—	
High		0.76	0.33, 1.73	0.506
**Baseline MTV**	139			
Low		—	—	

Characteristic	N	HR[1]	95% CI[1]	p-value
High		3.01	0.41, 22.3	0.282
**Baseline TLG**	116			
Low		—	—	
High		2.08	0.74, 5.84	0.164
**Delta SUV max**	136			
Low		—	—	
High		0.25	0.06, 1.06	0.060
**Delta TLG**	113			
Low		—	—	
High		0.24	0.07, 0.83	**0.024**
**Delta MTV**	137			
Low		—	—	
High		0.38	0.16, 0.89	**0.027**

[1] HR = Hazard Ratio, CI = Confidence Interval
